# Interventions to Prevent Obesity in Mexican Children and Adolescents: Systematic Review

**DOI:** 10.1007/s11121-021-01316-6

**Published:** 2021-11-02

**Authors:** Magaly Aceves-Martins, Lizet López-Cruz, Marcela García-Botello, Yareni Yunuen Gutierrez-Gómez, Carlos Francisco Moreno-García

**Affiliations:** 1 The Rowett Institute of Nutrition and Health, University of Aberdeen, Foresterhill, Aberdeen, AB25 2ZD UK; 2 Universidad Europea del Atlántico, Parque Científico Y Tecnológico de Cantabria, C/Isabel Torres 21, 39011 Santander, Spain; 3 Universidad de Monterrey, Zona Valle Poniente, Av. Ignacio Morones Prieto 4500, 66238 San Pedro Garza García, N.L Mexico; 4 Nutrition and Wellbeing Department, Tecnológico de Monterrey, Col. Ejidos de Huipulco, Tlalpan, Mexico City, Mexico; 5 School of Computing, Robert Gordon University, Garthdee House, Garthdee Road, Aberdeen, AB10 7QB Scotland, UK

**Keywords:** Obesity, Prevention, Children, Adolescents, Mexico

## Abstract

The prevalence of overweight and obesity has been rising among Mexican children and adolescents in the last decades. To systematically review obesity prevention interventions delivered to Mexican children and adolescents. Thirteen databases and one search engine were searched for evidence from 1995 to 2021. Searches were done in English and Spanish to capture relevant information. Studies with experimental designs, delivered in any setting (e.g., schools or clinics) or digital domains (e.g., social media campaigns) targeting Mexican children or adolescents (≤ 18 years) and reporting weight outcomes, were included in this review. In addition, the risk of bias was appraised with the Effective Public Health Practice Project Quality Assessment Tool. Twenty-nine studies with 19,136 participants (3–17 years old) were included. The prevalence of overweight and obesity at baseline ranged from 21 to 69%. Most of the studies (89.6%) were delivered in school settings. The duration ranged from 2 days to 3 school years, and the number of sessions also varied from 2 to 200 sessions at different intensities. Overall, anthropometric changes varied across studies. Thus, the efficacy of the included studies is heterogeneous and inconclusive among studies. Current evidence is heterogeneous and inconclusive about the efficacy of interventions to prevent obesity in Mexican children and adolescents. Interventions should not be limited to educational activities and should include different components, such as multi-settings delivery, family inclusion, and longer-term implementations. Mixed-method evaluations (including robust quantitative and qualitative approaches) could provide a deeper understanding of the effectiveness and best practices.

**Supplementary Information:**

The online version contains supplementary material available at 10.1007/s11121-021-01316-6.

## Introduction

The prevalence of overweight and obesity is a major international public health problem and has nearly doubled in the last three decades, especially among children and adolescents (Global Obesity Observatory, 2019). Excess body fat in children and adolescents can lead to various clinical conditions and psychosocial disorders that might significantly reduce their quality of life. Moreover, children and adolescents with obesity are likely to maintain their weight status into adulthood, increasing their risk of developing chronic diseases, contributing to increased morbidity and premature mortality (WHO, 2012; Wang & Lim, 2012).

Mexico is an upper-middle-income Latin-American country where obesity levels have been increasing alarmingly in the last decades (Aceves-Martins et al., 2016a; Astudillo, 2014). Specifically, overweight and obesity rates have increased in the < 18 years old population. According to the latest results from the National Health and Nutrition Survey (ENSANUT, 2018), it is estimated that 8.2% of infants (0–4 years), 35.6% of school-age children (5–11 years), and almost 40% of adolescents (12–19 years) have overweight or obesity in Mexico. In addition, the Global Obesity Observatory (2019) suggests that Mexico has one of the highest prevalence of obesity among children and adolescents worldwide. Furthermore, according to the Organisation for Economic Co-operation and Development estimations, obesity rates will continue to rise in Mexico if no effective strategies are implemented (OECD, 2017). Likewise, some economic models have estimated that childhood obesity in Mexico's economic impact between 2006 and 2050 will be much higher than what the health care system can stand, jeopardising the general population's health care and wellbeing (Garduño-Espinosa et al., 2008; Ortega-Cortés, 2014).

Most of the published systematic reviews on childhood obesity prevention include only English publications (Ash et al., 2017; Liu et al., 2019; Tamayo et al., 2021; Ward et al., 2017) or evidence from high-income countries (Tamayo et al., 2021; Wang et al., 2015), excluding valuable evidence from non-English speaking low- or middle-income countries such as Mexico. The “Childhood and adolescent Obesity in MexicO: evidence, challenges and opportunities” (COMO) Project intend to synthesise and use data to comprehend the extent, nature, effects, and costs of childhood and adolescent obesity in Mexico (Aceves-Martins, 2021a, b, c). This systematic review is part of the COMO project, and it aims to identify and evaluate studies implemented in Mexico to prevent obesity among children and adolescents (< 18 years).

## Methods

This project’s systematic review is registered in The International Prospective Register of Systematic Reviews (Registration number CRD42019154132) (PROSPERO, 2021). In addition, this review is reported according to Preferred Reporting Items for Systematic Reviews and Meta‐analyses guidelines (PRISMA, 2021). The research question and inclusion/exclusion criteria were established following the Population, Intervention, Comparison, Outcomes, Study design (PICOS) framework.

### Electronic Searches

A sensitive search was developed to include index terms, free-text words, abbreviations, and synonyms to combine the key concepts for this review. Terms such as “overweight,” “obesity,” “child,” “adolescent,” “intervention,” “program,” and “Mexico” were included in the strategy with different term variation/synonyms and Boolean connectors to capture relevant publications. The databases searched included MEDLINE, EMBASE, the Cochrane Library, Global Health Library, LILACS, CINAHL, CAB abstracts, ERIC, PsycINFO, ScienceDirect, Scopus, AGRICOLA, and SciELO Citation Index. Also, the search engine Google Scholar was used to identify relevant studies. When possible, searches were also done in Spanish to capture relevant references. Conference abstracts and poster presentations were included if the inclusion criteria were met. Also, reference lists of the included studies were scrutinised for additional publications, and experts in the field were contacted for additional relevant reports. Searches were done in January 2020 and updated in January 2021.

### Selection Criteria

Reports from 1995 onwards were included in this review to focus on information conducted under current epidemiological and environmental circumstances of child and adolescent obesity in Mexico. All searches were restricted to English, Spanish, or Portuguese language publications to capture reports from the most widespread languages spoken in the Americas. Following the PICOS framework, our inclusion/exclusion criteria were:

#### Population

Children and adolescents from zero to 18 years old (mean age at the start of the study) from any ethnicity or gender living in Mexico were included. Studies that involved parents, caregivers, or related stakeholders (e.g., teachers or health carers) were included only if the outcomes were measured in children or adolescents. Studies evaluating the treatment (i.e. only including participants with obesity) were excluded from this review. Mexican children living in different countries were excluded to better conceptualise the obesity problem within their sociodemographic characteristics, avoiding confounding information inherent to the migration phenomena. Likewise, studies that analysed children's severe conditions (e.g. HIV, cancer, down syndrome), premature babies and pregnant adolescents were excluded.

#### Intervention

Obesity prevention or lifestyles interventions delivered among Mexican children or adolescents were considered.

#### Outcomes

Since weight and weight-related measures are indispensable for the evaluation effectiveness of interventions related to child and adolescent overweight and obesity (Green et al., 2012), weight-related outcomes (i.e., weight, BMI, or BMI z-score change) were considered in this review as primary outcomes. Because of the studies' nature, any other outcome related to lifestyle changes (i.e., dietary, PA, behavioural outcomes) was also recorded as secondary outcomes.

#### Study Design

Any experimental or quasi-experimental studies designs were considered. In addition, interventions delivered in any setting (e.g. home-based, school-based, clinic-based, community-based, leisure centres) or digital domains (e.g. social media interventions) were considered.

#### Data Extraction

Titles, abstracts, and relevant full-texts were screened by two reviewers (LL, MGB) and 100% checked by a third reviewer (MA-M). Two reviewers (MA-M and LLC) extracted data from intervention studies independently. In case of any disagreement, a third author was contacted (YG).

A data extraction form was created based on the Effective Public Health Practice Project Quality Assessment Tool (EPHPP, 2010) for quantitative studies and the PICOS framework. The template for intervention description and replication (TIDieR, 2021) checklist items were also included in the extraction form. Critical components of the interventions were extracted and categorised as a) Nutritional (i.e., studies including diet prescriptions or nutritional advice); b) PA (i.e., studies including PA practise or PA advice); c) behavioural or psychological (i.e., studies including counselling, or behavioural therapy); and d) environmental changes (i.e., environment changes to promote a weight change among participants). Any strategy or framework used in the design of the interventions was also recorded.

### Quality and Risk of Bias Assessment

Following the Cochrane Handbook's recommendation about systematic reviews of health promotion interventions (Armstrong et al., 2011), the EPHPP (2010) Assessment Tool for Quantitative Studies was used. This tool produces an overall methodological rating (i.e., strong, moderate, or weak evidence) and comprises eight categories: selection bias; study design; confounders; blinding; data collection methods; withdrawals and drop-outs; intervention integrity; and analysis. According to the quality assessment tool’s guidelines for each category, the scores were added, producing a global rating. In addition, the funding source and reported conflicts of interest were also extracted. Two reviewers (MA-M and LLC) evaluated each included study independently and then agreed. In case of any disagreement, a third author was contacted (YG).

### Data Synthesis

A meta-analysis was not feasible because of the heterogeneity among the included studies and the lack of outstanding quality Randomised Controlled Trials [RCTs]. Hence, a narrative synthesis was conducted. The data obtained from the included studies were narratively synthesised, and relevant characteristics were tabulated. According to the type of study (i.e. studies including a control group and cohort studies without control groups), results were reported in the text. In addition, textual descriptions of studies and reported statistical significance were recorded and tabulated. Quality assessment was included in the synthesis.

## Results

**Fig. 1:**
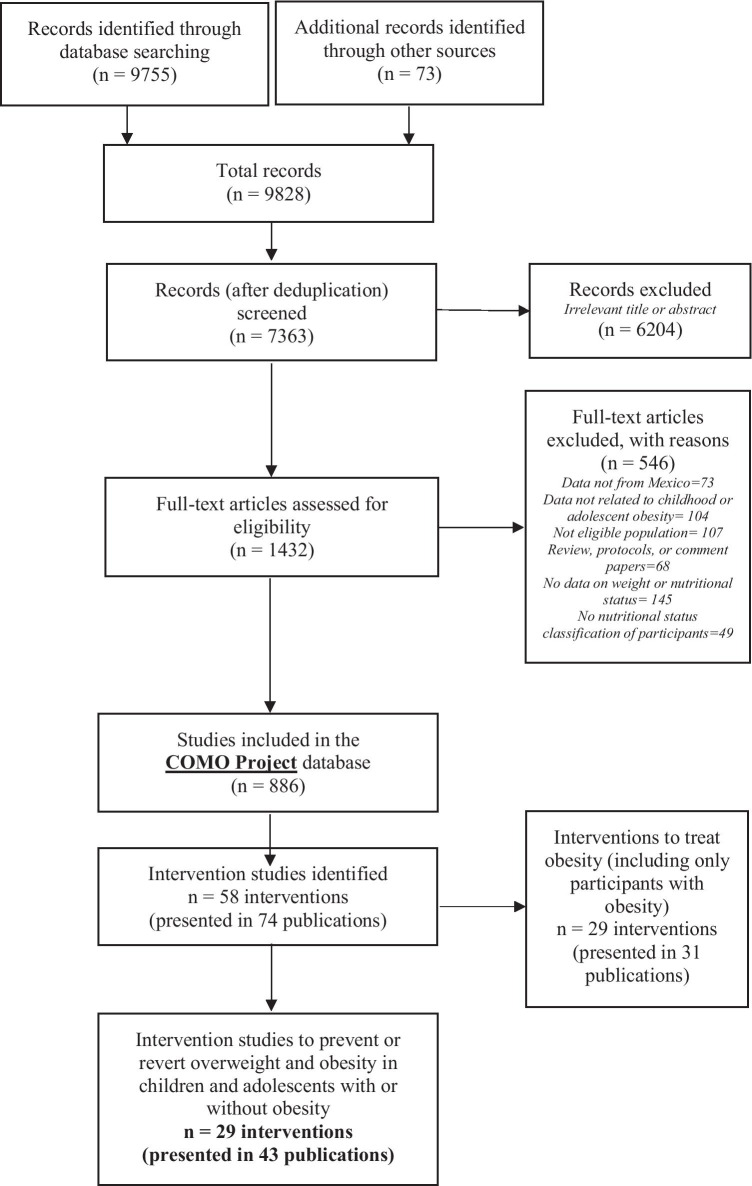
PRISMA flowchart

Our searches identified 9828 references, from which 1432 were retrieved for full-text review. Thus, overall, 886 references were included in the COMO database. From these, 58 studies (presented in 74 publications) were identified. From these, 29 studies (presented in 43 publications) met the inclusion criteria **(**Fig. 1). Nearly half of the studies (14/29) were published in Spanish, and the rest in English. Also, less than half (12/29) were published in Mexican journals.

From the 29 studies included, four (13.7%) (Levy et al., 2012a, b; Macias et al., 2014; Mejia et al., 2016; Morales-Ruán et al., 2014) were conference abstracts. Sixteen studies (55.2%) included control groups (seven [24.1%] were RCTs, one [3.4%] pilot RCT, eight [27.5%] were controlled trials), and 13 (44.8%) were cohorts (11 [37.9%] were single cohorts, and two [6.8%] were cohort analytic, including two intervention groups) (Table 1).

**Table 1:** Principal characteristics of included studies

**STUDY ID** Study design	**Setting characteristics** (City or Town, Federal State, Setting, Year of implementation)	**Participant's characteristics**	**Intervention's key characteristics**	**Nutritional component**	**PA component**	**Behavioural component**	**Environmental changes**	**Weight reported outcomes**
Alvirde-Garcia et al. (2013) Randomised Control Trial	Tenango del Valle and Santa Cruz de Atizapan (State of Mexico) Two public schools from semi-rural communities 2007–2010	**Total initial sample:** 2682 **Female (%):** 50.2 **Mean (SD) age:** 9 (1.7) years **Baseline prevalence of OW + OB (%):** 38	**Duration:** 3 school years **Follow-up period:** NR **Intensity and Frequency:** 15 sessions **Delivered by:** Teachers (previously trained by the research staff) **Overall Scope:** Educational intervention to change lifestyles through didactic material and family-based activities (replication of Child and Adolescent Trial for Cardiovascular Health originally from the US)	☐	☐	NR	☐	After three years, the study resulted in a lower BMI increase but no significant weight change
Arroyo and Carrete (2018) Cohort (one group before and after)	Toluca (State of Mexico) Public schools from middle-SES neighbourhoods 2014	**Total initial sample:** 98 **Female (%):** 52 **Mean (SD) age:** 11.8 (0.5) years **Baseline prevalence of OW + OB (%):** 21.2	**Duration:** 3 months **Follow-up period:** NR **Intensity and Frequency:** 4 conferences + weekly homework + 2 optional conferences for parents **Delivered by:** Medical doctors and a nutritionist **Overall Scope:** Educational intervention intended to promote healthy eating practices	☐	NR	☐	NR	No weight or BMI changes were reported at the end of the study
Bacardi-Gascon and Jiménez-Cruz (2012) Randomised Control Trial	Tijuana (Baja California) Two public and two private schools from similar SES 2008–2010	**Total initial sample:** 532 **Female (%):** 48.9 **Mean (SD) age:** 8.4 (NR) years **Baseline prevalence of OW + OB (%):** 45.3	**Duration:** 6 months + 18 months **Follow-up period:** 18 months, but a follow-up program was continued during this time **Intensity and Frequency:** 3 sessions (60 min each) with school board and teachers + 8 sessions (30 min per week × 8 weeks) with children + 4 sessions (60 min per month × 4 months) with parents **Delivered by:** Nutrition (graduate students and professionals) and PA professionals **Overall Scope:** Educational intervention that implemented changes in the school curricula and included school board, teachers, and parents' involvement	☐	☐	NR	☐	At six months, there were significant BMI differences between the control and intervention groups
Balas-Nakash et al. (2010) Cohort analytic (two groups before and after)	Toluca (State of Mexico), Two public schools serving middle-SES children and that had suitable playgrounds for activities 2008	**Total initial sample:** 319 **Female (%):** 59.6 **Mean (SD) age:** 10 (NR) years **Baseline prevalence of OW + O B (%):** 43.9	**Duration:** 3 months **Follow-up period:** NR **Intensity and Frequency:** 60 afterschool sessions (20 min for children in routine A and 40 min for children in routine B 5 days a week) **Delivered by:** Certified physical education trainer **Overall Scope:** PA practice, including two different routines at different intensities	NR	☐	NR	NR	Anthropometric measures (e.g., fat mass percentage, BMI) significantly decreased in routine B children. In addition, the prevalence of overweight and obesity decreased in both groups significantly
Benitez-Guerrero et al. (2016) Controlled trial	Tepic (Nayarit) Twelve public primary schools from an urban area Year of implementation NR	**Total initial sample:** 368 **Female (%):** 48.3 **Range age:** 9–11 years **Baseline prevalence of OW + OB (%):** 42.3	**Duration:** 3 months **Follow-up period:** NR **Intensity and Frequency:** NR **Delivered by:** NR **Overall Scope:** Educational intervention intended to promote healthy eating PA	☐	☐	NR	NR	Girls in the intervention group reduced underweight and overweight prevalence, reflecting a positive effect of the intervention. No effect was observed in boys
Caballero-Garcia et al. (2017) Cohort (one group before and after)	Chilpancingo (Guerrero) Puerto Vallarta (Jalisco), Coatetelco (Morelos) and Hermosillo (Sonora) Four public schools from medium- and low-SES. Some schools included indigenous and working children 2006–2008	**Total initial sample:** 1031 **Female (%):** 50.1 **Mean (SD) age:** 10 (NR) years **Baseline prevalence of OW + OB (%):** 27.6	**Duration:** 5 months **Follow-up period:** NR **Intensity and Frequency:** 20 educational sessions (60 min once a week) **Delivered by:** Education facilitator (external school staff, undergraduate/graduate) and research team **Overall Scope:** Educational intervention to promote healthy eating	☐	NR	NR	NR	Overall, the weight or BMI reduction varied across sites. There was a reduced prevalence of obesity in Sonora and a reduced prevalence of overweight in Morelos, Jalisco and Sonora
Costa-Urrutia et al. (2019) Cohort analytic (two groups before and after)	Hermosillo, Punta Chueca and Bahía de Lobos (Sonora) Four general urban schools (from Hermosillo, the capital city) and two indigenous schools, Seris (from Punta Chueca) and Yaquis (from Bahía de Lobos) 2016	**Total initial sample:** 320 **Female (%):** 49 **Mean (SD) age:** 8.2 (2.3) years **Baseline prevalence of OW + OB (%):** 39.4	**Duration:** 3 months **Follow-up period:** NR **Intensity and Frequency:** 36 PA sessions (of 60 min) + 24 PA sessions (of 45 min) + 12 workshops (of 50 min) + 3 workshops for parents (length NR) **Delivered by:** Physical education teacher, nutritionist, and phycologists (previously trained), and teachers supported **Overall Scope:** PA practice, health education, parent involvement and school meals provision	☐	☐	☐	☐	BMI decreased significantly in children with overweight and obesity. Mestizos under group 1 (PA, health education and parent involvement components) increased BMI, whereas those in group 2 (group 1 intervention + school meals) decreased it. Seris increased BMI, Yaquis increased BMI significantly. Concerning ethnic groups, Mestizos and Seris decreased their BMI values, but not significantly. Yaquis increased their BMI values, and such an amount of increase decreases with age
Cruz-Bello et al. (2019) Cohort (one group before and after)	San Cristóbal Tecolit Municipality and Zinacantepec Municipality (State of Mexico) One public high school Year of implementation NR	**Total initial sample:** 32 **Female (%):** 43.4 **Mean (SD) age:** 15.6 (1.3) years **Baseline prevalence of OW + OB (%):** 43.7	**Duration:** 4 months **Follow-up period:** 3 months after the intervention **Intensity and Frequency:** 40 sessions (60 min each) **Delivered by:** Trained Nurses **Overall Scope:** Educational intervention aiming to promote healthy eating and PA open classes	☐	☐	☐	NR	No weight or BMI changes were reported at the end of the study
Elizondo-Montemayor et al. (2014) Cohort (one group before and after)	Monterrey (Nuevo Leon) Five private high schools in the urban area 2011–2012	**Total initial sample:** 554 **Female (%):** 48.1 **Range age:** 14–17 years **Baseline prevalence of OW + OB (%):** 25	**Duration:** 1 school year **Follow-up period:** NR **Intensity and Frequency:** Weekly updates on social media channels + 1 (optional) PA session **Delivered by:** Medical doctor, nutritionists, and medical and nutrition interns. Teachers continuously invited the students to go to these means to receive the counselling **Overall Scope:** Social media health promotion activities (weekly updates), conferences and PA open classes	☐	☐	NR	NR	No significant differences were found in the prevalence of obesity and overweight at the baseline and end of the study. However, there was a significant increase in BMI and fat percentage among female participants
Gatica-Dominguez et al. (2019) Controlled trial	Tlaltizapan, Zacatepec and Galeana (Morelos) Four elementary public schools in Tlaltizapán town (intervention) and four primary schools in Galeana town (control) 2010–2013	**Total initial sample:** 214 **Female (%):** 48.6 **Range age:** 8–14 years **Baseline prevalence of OW + OB (%):** 35.6	**Duration:** 3 school years **Follow-up period:** NR **Intensity and Frequency:** NR **Delivered by:** NR **Overall Scope:** Intervention, including dietary, PA, and social participation components. However, the identified study only describes the PA component. Strategies targeted children, parents, teachers, educational authorities, community leaders and local government authorities	☐	☐	NR	NR	No weight or BMI changes were reported at the end of the study
Macias et al. (2014) (Abstract) Randomised Control Trial	Leon (Guanajuato) One elementary school Year of implementation NR	**Total initial sample:** 135 **Female (%):** NR **Mean (SD) age:** NR **Baseline prevalence of OW + OB (%):** 24.6	**Duration:** 6 months **Follow-up period:** 6 months **Intensity and Frequency:** 48 sessions (2 sessions per week) **Delivered by:** NR **Overall Scope:** Educational intervention designed for children about PA and nutrition in elementary schools based on theory to change habits	☐	☐	NR	NR	At one year of follow-up, more children with overweight and obesity were reported in the control group than the intervention group
Martinez-Andrade et al. (2014) Pilot—Randomised Control Trial	Mexico City (Mexico City) Four public primary healthcare clinics 2012 (Cespedes et al. 2012)	**Total initial sample:** 1406 **Female (%):** 47.4 **Mean (SD) age:** 3.4 (0.8) years **Baseline prevalence of OW + OB (%):** 55.9	**Duration:** 1.5 months **Follow-up period:** 6 months **Intensity and Frequency:** 6 sessions (120 min weekly sessions) + 1 educational session on PA (90 min) + 1 socialising session (30 min) **Delivered by:** Nutritionist, nurse and health educator **Overall Scope:** Intervention based on motivational counselling to change eating behaviours and PA was delivered. Mexican adapted version of the "High Five for Kids" intervention from the US	☐	☐	☐	NR	When using an intention to treat analysis, no BMI changes were found at either 3 or 6 months
Mejia et al. (2016) (Abstract) Randomised Control Trial	Tamaulipas City, (Tamaulipas) Two elementary schools (no further information provided) Year of implementation NR	**Total initial sample:** NR **Female (%):** NR **Mean (SD) age:** NR **Baseline prevalence of OW + OB (%):** 56	**Duration:** 4 months **Follow-up period:** NR **Intensity and Frequency:** NR **Delivered by:** Unclear **Overall Scope:** Educational intervention named a culturally sensitive health education model to prevent child obesity targeting teachers, parents, and children	☐	☐	NR	☐	The upward trend of BMI was reversed among children with overweight/obesity in the intervention group, while the upward trend of the BMI in the control group continued to increase. However, these changes were not significant after four months
Padilla-Raygoza et al. (2013) Randomised Control Trial	Celaya (Guanajuato) Elementary public schools Year of implementation NR	**Total initial sample:** 301 **Female (%):** NR **Range age:** 6–13 years **Baseline prevalence of OW + OB (%):** 66	**Duration:** 4 months **Follow-up period:** NR **Intensity and Frequency:** 80 walking sessions (1 session 30 min 5 days a week for four months) + 8 sessions to mothers **Delivered by:** NR **Overall Scope:** The intervention included 30 min of daily PA and teaching in selecting and preparing meals for the children's mother	☐	☐	NR	NR	After the four months, there were non-significant differences in the prevalence of overweight or obesity among groups. However, weight and BMI were significantly lower in the intervention compared to the control group
Perichart-Perera et al. (2008) Cohort (one group before and after)	Santiago de Queretaro (Queretaro) Two public schools from urban areas Year of implementation NR	**Total initial sample:** 360 **Female (%):**53.1 **Range age:** 8–14 years **Baseline prevalence of OW + OB (%):** 42.2	**Duration:** 4 months **Follow-up period:** NR **Intensity and Frequency:** 80 sessions (20 min 5 days a week) + 16 sessions (minimum of 30 min per week of teacher's advice) **Delivered by:** Physical educator, teachers, nutritionists, and paediatric nurses **Overall Scope:** PA practice and food orientation intervention for schoolchildren promoted by teachers to increase PA in schoolchildren and provide messages that helped achieve a healthy balance	☐	☐	NR	NR	After the intervention, a non-significant reduction in waist circumference, BMI was reported. Children who had overweight and obesity at baseline had a higher risk score than those with normal BMI. However, this score did not decrease significantly after the intervention
Polo-Oteyza et al. (2017) Cohort (one group before and after)	Toluca Valley, including Metepec, Ocoyoacac, Huixquilucan and Lerma (State of Mexico) 5 public schools from rural and urban areas 2013–2014 (Palacios-González et al. 2015)	**Total initial sample:** 1888 **Female (%):** NR **Range age:** 6–11 years **Baseline prevalence of OW + OB (%):** 31.9	**Duration:** 1 school year **Follow-up period:** NR **Intensity and Frequency:** 200 PA sessions (30-min routine to be performed five days a week) **Delivered by:** Teachers, PA teachers, a medical doctor, paediatric nurses, nutritionists, and research assistants **Overall Scope:** PA intervention designed and implemented by teachers in charge of the physical education activities in public schools	×	☐	NR	NR	No significant changes were found in BMI or waist circumference after the intervention, even after correcting children's growth
Ponce et al. (2016) Controlled trial	Mexicali (Baja California) Secondary schools (No further information provided) Year of implementation NR	**Total initial sample:** 418 **Female (%):** 54.3 **Range age:** 11–15 years **Baseline prevalence of OW + OB (%):** 40.6	**Duration:** 2 months **Follow-up period:** NR **Intensity and Frequency:** 6 sessions (totalling 28 h) **Delivered by:** NR **Overall Scope:** Educational intervention which promoted healthy dietary lifestyles	☐	NR	NR	NR	There was a significant decrease in weight and BMI in the intervention group compared to controls. However, the prevalence of overweight and obesity was higher in the control group and even higher in males from the control group
Radilla-Vazquez et al. (2019) Controlled trial	Mexico City (Mexico City) 16 public secondary schools Year of implementation NR	**Total initial sample:** 2368 **Female (%):** 49.7 **Mean (SD) age:** 11.8 (0.5) years **Baseline prevalence of OW + OB (%):** 39.2	**Duration:** 3 school years **Follow-up period:** NR **Intensity and Frequency:** NR **Delivered by:** Medical doctors and a provider of social nutrition service from the selected schools and one of the professional practices in psychology, with the support of a social worker for each school **Overall Scope:** Educational intervention using comic-type printed materials to promote healthy lifestyles and food choices	☐	NR	☐	NR	After the intervention, the prevalence of obesity and overweight decreased in the intervention group, while the control group remained similar
Ramirez-Lopez et al. (2005) Controlled trial	24 communities from 17 municipalities (Sonora) Schools from considered communities, including both rural and urban 2002–2003	**Total initial sample:** 610 **Female (%):** NR **Mean (SD) age:** 8.5 (1.3) years **Baseline prevalence of OW + OB (%):** 41.1	**Duration:** 1 school year **Follow-up period:** NR **Intensity and Frequency:** 180 breakfasts (breakfast provision 5 days a week for 9 months) **Delivered by:** Social workers or teachers **Overall Scope:** School breakfast programme, plus and education and PA intervention	☐	NR	NR	NR	No significant differences were found between the two groups in height/age, BMI, and fat percentage. In addition, the prevalence of overweight or obesity did not change after the intervention
Rios-Cortazar et al. (2013) Cohort (one group before and after)	Mexico City (Mexico City) One public elementary school 2008–2011	**Total initial sample:** 232 **Female (%):** NR **Mean (SD) age:** NR **Baseline prevalence of OW + OB (%):** 34.4	**Duration:** 3 school years **Follow-up period:** NR **Intensity and Frequency:** NR **Delivered by:** NR **Overall Scope:** Health promotion intervention using children's narrative and actions to construct a school environment that promotes health	☐	☐	NR	NR	The prevalence of overweight decreased significantly
Vazquez et al. (2017) Cohort (one group before and after)	Cd. Victoria (Tamaulipas) Public secondary school Year of implementation NR	**Total initial sample:** 54 **Female (%):** 48.9 **Range age:** 11–14 years **Baseline prevalence of OW + OB (%):** 57.4	**Duration:** 2 days **Follow-up period:** NR **Intensity and Frequency:** 2 sessions (90 min each) **Delivered by:** Nurses **Overall Scope:** Educational nursing intervention following the Clinical Practice Guideline on Nursing Interventions to prevent overweight and obesity in children and adolescents in the first level of care	☐	NR	NR	NR	Baseline anthropometric data presented only. Weigh changes not reported at the end of the intervention
Rodriguez-Ventura et al. (2018) Pilot—Cohort (one group before and after)	Mexico City (Mexico City) Paediatrics department of a public hospital Year of implementation NR (Rodriguez-Ventura et al. 2014)	**Total initial sample:** 55 **Female (%):** 50 **Mean (SD) age:** 13.5 (NR) years **Baseline prevalence of OW + OB (%):** 55.6	**Duration:** 3–4 months **Follow-up period:** NR **Intensity and Frequency:** 3–4 sessions (single monthly visit) + 2 workshops **Delivered by:** Registered Dietitian, Paediatric Endocrinologist, Psychologists (if necessary) **Overall Scope:** Clinical and nutritional education intervention "*Sacbe*" (Mayan word that means "the white way") based on the Diabetes Prevention Programme (originally from the US)	☐	☐	☐	NR	Using an intention to treat analysis, obesity prevalence and BMI z-scores decreased significantly
Safdie et al. (2013a, b) Randomised Control Trial	Mexico City (Mexico City) Forty public elementary schools from low-SES children receiving benefits from the Federal School Breakfast Program 2006–07 and 2007–08 school years (Aburto et al. 2011; Bonvecchio et al. 2014; Bonvecchio-Arenas et al. 2010; Safdie et al.^2014^)	**Total initial sample:** 886 **Female (%):** 50 **Mean (SD) age:** 9.7 (0.7) years **Baseline prevalence of OW + OB (%):** 43	**Duration:** 2 school years **Follow-up period:** NR **Intensity and Frequency:** Implementation of both interventions varied and depend on the willingness of principals, teachers, and school staff **Delivered by:** Physical Education teachers **Overall Scope:** Intervention focused on improving nutrition and PA norms at the schools and limited existing school infrastructure and resources. The "plus intervention" implemented all the primary intervention components and included additional financial investment and human resources	☐	☐	☐	☐	The prevalence of overweight and obesity in children changed across the evaluation period by type of intervention group. There was a BMI reduction in children in control schools from baseline to months 7, 11 and 18 and increased BMI in primary intervention schools from baseline to 7, 11 and 18 months. Overall, the interaction between intervention duration and type for BMI was significant. There was a significant difference in BMI between baseline and seven months, between 7 and 11 months, and between baseline vs 18 months
Salazar-Vazquez et al. (2016) Controlled trial	Durango City (Durango) One private school 2011	**Total initial sample:** 54 **Female (%):**43 **Mean (SD) age:** 12.5 (2) years **Baseline prevalence of OW + OB (%):** 35.4	**Duration:** 1 school year **Follow-up period:** NR **Intensity and Frequency:** 2 measurement sessions + adherence of each participant **Delivered by:** NR **Overall Scope:** Intervention designed to reduce the eating rate and foster awareness of the onset of the satiety reflex. Study participants received a 30-s-period portable hourglass used to pace bites' timing during meal consumption	☐	NR	☐	NR	Results are presented by adhering vs not adhering to groups. The BMI and BMI z scores significantly decreased after the first semester and second semester, the adhering group. In contrast, the BMI in the nonadherent and the control groups significantly increased after one year. In addition, the prevalence of participants with overweight and obesity adhering to the study decreased significantly at six months and 12 months
Saucedo-Molina et al. (2018) Pilot—Cohort (one group before and after)	Hidalgo City (Hidalgo) Public high school from an urban area and pupils from various SES 2014	**Total initial sample:** 368 **Female (%):** 58.1 **Mean (SD) age:** 16.4 (NR) years **Baseline prevalence of OW + OB (%):** 50	**Duration:** 5 days **Follow-up period:** 6 months **Intensity and Frequency:** 4 sessions (1 h each) + 4 PA sessions (1 h each) + 1 final workshop (90-min) **Delivered by:** Undergraduate nutrition degree students **Overall Scope:** Educational prevention intervention comprising prevention of eating risky and sedentary behaviours based on The Body Project and the Eating, Aesthetic, Feminine Models and Media Programme, comprised five activity-based sessions, delivered on five consecutive days. Including enjoyable PA and three parallel workshops	☐	☐	☐	NR	A significant change in BMI distributions and a downwards trend was observed in students with overweight and obesity. In addition, the prevalence of overweight or obesity decreased among adhering participants compared with the non-adhering group
Levy et al. (2012a, b) Randomised Control Trial	125 municipalities (State of Mexico) 60 Public elementary schools were serving children that were beneficiaries of a school breakfast program 2010–2011 (Levy et al. 2011)	**Total initial sample:** 1019 **Female (%):** 50.6 **Range age:** 10–13 years **Baseline prevalence of OW + OB (%):** 35.4	**Duration:** 6 months **Follow-up period:** 6 months **Intensity and Frequency:** 6 sessions (workshops) + 4 sessions (once per week for four weeks puppet theatre) + approx. 48 sessions (PA twice per week sessions which gradually increased from 2 to 5 days) + 24 play activities at breaks + 2 sessions (workshops for teachers) + 1 session (1-h session for food store personnel) **Delivered by:** Nutritionists and health professionals (nurses and social workers previously trained), psychologists and educators and physical trainers and standardise health promoters **Overall Scope:** Multi-component intervention including a gradual decrease of the energy content of school breakfasts, a gradual regulation of food offered within the school, gradual adherence to the PA intervention and implementing an educational campaign	☐	☐	☐	☐	The probability of having obesity at the end of the intervention decreased in the intervention group while it increases in the control group. Thus, the intervention had a small but significant effect on reducing the probability of shifting from the overweight to the obesity category after six months of intervention. In addition, this study also documented a decreasing effect on the shift from the normal to the overweight categories during six months of intervention
Vega et al. (2019) Controlled trial	Mexico City (Mexico City) 16 secondary schools Year of implementation NR	**Total initial sample:** 2368 **Female (%):** 49.7 **Mean (SD) age:** 12.1 (0.5) years **Baseline prevalence of OW + OB (%):** 39.1	**Duration:** 3 school years **Follow-up period:** NR **Intensity and Frequency:** NR **Delivered by:** School doctors, science, and physical education teachers **Overall Scope:** Educational intervention on Food orientation was provided to students and parents using educational materials	☐	NR	☐	NR	After the intervention, the prevalence of obesity decreased significantly in the intervention group
Vilchis-Gil et al. (2016) Controlled trial	Mexico City (Mexico City) Four elementary schools (two public and two private) from a middle SES area 2013–2014 (Vilchis-Gil et al. 2018)	**Total initial sample:** 407 **Female (%):** 46.9 **Mean (SD) age:** 8 (1.2) years **Baseline prevalence of OW + OB (%):** 49.2	Educational intervention for parents and children, including sessions to promote healthy eating habits and exercise. A website and text messages to reinforce the information were sent to parents' mobile phones, reinforcing the information. Also, workshops and visits to museums were part of the intervention	☐	☐	☐	NR	After the intervention, the intervention group decreased the BMI z-score, while the control group increased it
Zacarias et al. (2019) Cohort (one group before and after)	Montenegro (Queretaro) Low SES community 2016–2018	**Total initial sample:** 57 **Female (%):** NR **Mean (SD) age:** 8.1 (1.5) years **Baseline prevalence of OW + OB (%):** 69	**Duration:** 6 months **Follow-up period:** NR **Intensity and Frequency:** 6 sessions (1 monthly 90 min-session) **Delivered by:** Nutritionist **Overall Scope:** Intervention to improve the mother's knowledge and skills necessary to change children's food behaviour positively	☐	NR	☐	NR	After the intervention, children significantly reduced BMI z-score and waist circumference-height ratio

*SD* standard deviation; *OW* overweight; *OB* obesity; *SES* socioeconomic status; *min* minutes; *hr* hours; *PA* physical activity; *US* United States of America; *NR* not reported. ☐ = Component included ×  = Component not included. Intensity and Frequency were estimated from the reported data

**Fig. 2:**
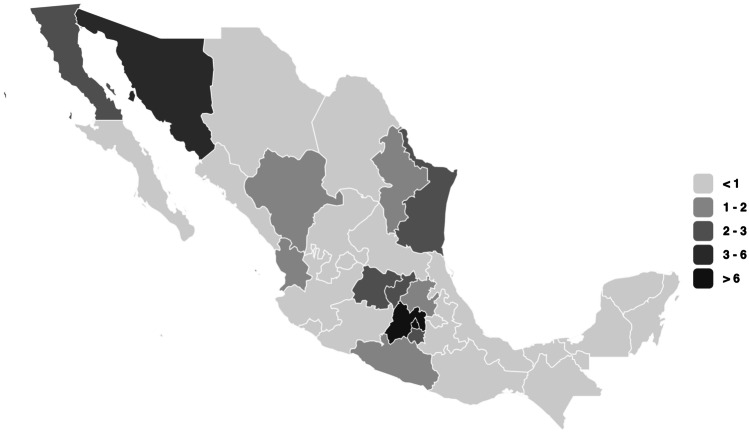
Map from the origin of the included interventions

Overall, this systematic review includes data from 19,136 participants (from 3 to17 years old) recruited from 13 Mexican states (out of 32) (Fig. 2). Only Caballero-García et al. (2017) implemented a multi-site intervention that included pupils from different states across Mexico. The prevalence of overweight and obesity at baseline ranged from 21 to 69% across included studies. All the studies targeted and included both males and female participants. Most of the studies (89.6%) were delivered in school settings, two (Martínez-Andrade et al., 2014; Rodriguez-Ventura et al., 2018) in public clinics, and one (Zacarías et al., 2019) in a community setting. One study (Martinez-Andrade et al., 2014) was delivered among a preschool population, 22 studies among a school-age population (6–12 years), and six among adolescents (13–18 years). The principal characteristics of the included studies are presented in Table 1.

The design and implementation of the included studies varied widely. Some studies (13; 44.8%) included educational interventions, some others (5; 17.2%) included PA practise as an intervention, three (10.3%) included both educational intervention and PA practise, and one (3.4%) incorporated a school breakfast provision besides an educational intervention and PA practise. Elizondo-Montemayor et al. (2014) delivered a social media campaign plus a non-mandatory PA masterclass as part of the study. Ríos-Cortázar et al. (2013) used children's narratives to reconstruct the school's environment towards creating a healthier school atmosphere. Martínez-Andrade et al. (2014) and Salazar Vázquez et al. (2016) included solely motivational counselling. Rodriguez-Ventura et al. (2018) included a multidisciplinary clinical intervention. Zacarías et al. (2019) delivered a community intervention aiming to change children's weight but intervening mothers. However, this was not the only study including parents or other family members. Twelve studies (41.3%) included parents or siblings as active participants of the activities delivered to children (Table 1).

Overall, the components of the included studies also varied. Most of the studies (26; 89.6%) included a nutritional or dietary component. Some studies (17; 58.6%) included a PA component, and less (13; 44.8%) included behavioural or physiological components. Few studies (6; 20.7%) considered environmental or setting changes (e.g., modifications to the school food stores or improving the school’s infrastructure) (Table 1). Only Costa-Urrutia et al. (2019), Safdie et al. (2013a, b) and Levy et al. (2012a, b) included all the components considered in this review (i.e. considered in the studies a nutritional, PA, behavioural and an environmental change). The studies’ duration varied, ranging from 2 days to 3 school years. The frequency and intensity of the interventions (calculated from the reported data) also varied from 2 to 200 sessions with different intensities (Table 1). Further details on the components and characteristics of each study are provided in Supplementary Information 1.

Some studies (9; 31.0%) reported using a framework or theory during the intervention’s design and implementation: Bacardí-Gascon and Jiménez-Cruz (2012) used Bronfenbrenner's Ecological Model; Gatica-Domínguez et al. (2019) used the Booth’s Eco-social Model; Safdie et al. (2013a, b) used the Ecological Principles summed to a Theory of Planned Behaviour, Social Cognitive Theory, and a Health Belief Model; Zacarías et al. (2019) used the Theory-Informed Model, Social Cognitive Theory and Interpersonal Models; Martínez-Andrade et al. (2014) used the Chronic Care Model with Family-Centred Approach; Rodriguez-Ventura et al. (2018) used the Sociocultural and Precede-Proceed Models; Arroyo and Carrete (2018) used the Protection Motivation Theory; and Mejia et al. (2016) used the Psycho-Pedagogical Theory (including Social Cognitive Theory and Positive Psychology). Macias et al. (2014) reported using a “theory to change habits,” but no further detail was provided. Also, some studies reported using different delivery techniques. For instance, Costa-Urrutia et al. (2019), Saucedo-Molina et al. (2018) and Levy et al. (2012a, b) used participatory actions to deliver the activities or other psychoeducational strategies, such as peer-learning or empowerment, triggering technique and experience-based technique.

### Changes in Anthropometric Outcomes

Results were heterogeneous among studies that included a control group (*n* = 16). No statistically significant (*p* < 0.05) BMI or obesity prevalence changes were reported in 4/16 of the studies across the evaluation period or between groups (Alvirde-García et al., 2013; Gatica-Domínguez et al., 2019; Martínez-Andrade et al., 2014; Ramírez-López et al., 2005). Macias et al. (2014) and Mejia et al. (2016) reported that the upward BMI trend was reversed among children with overweight/obesity in the intervention group, while the control group's upward BMI trend continued to increase. However, no statistical significance test was presented. Some studies (9/16) found a significant statistical (*p* < 0.05) change in either weight or BMI (Padilla‐Raygoza et al., 2013; Ponce et al., 2016; Safdie et al., 2013a, b; Vilchis-Gil et al., 2016) or obesity prevalence (Bacardí-Gascon & Jiménez-Cruz, 2012; Perez-Morales et al., 2011; Radilla-Vazquez et al., 2019; Levy et al., 2012a, b; Vega et al., 2019) across the evaluation period and between groups. Benitez-Guerrero et al. (2016) reported only BMI effects among girls from the intervention group, but not in boys or the control group. Salazar-Vazquez et al. (2016) reported statistically significant (p < 0.05) BMI changes only in those participants from the intervention group that adhere to the intervention.

Results were also heterogeneous among cohort studies (*n* = 13). No statistically significant BMI or obesity prevalence changes were reported in 6/13 of the studies (Arroyo & Carrete, 2018; Cruz-Bello et al., 2019; Elizondo-Montemayor et al., 2014; Perichart-Perera et al., 2008; Polo-Oteyza et al., 2017; Vazquez et al., 2017). But some studies (5/13) reported significant reductions in BMI (Rodriguez-Ventura et al., 2018; Saucedo-Molina et al., 2018; Zacarias et al., 2019) or obesity prevalence (Balas-Nakash et al., 2010; Rios-Cortazar et al., 2013). Caballero-Garcia et al. (2017) was the only multi-site study (carried out in four different states of Mexico) and found that anthropometric changes varied depending on the site. Also, Costa-Urrutia et al. (2019) found that BMI decreased significantly depending on participants' baseline nutritional status and ethnicity.

Heterogeneity and inconsistency of results were also present when considering the different life stages. Weight-related outcomes did not differ or were evident in preschool children, school-aged children, or adolescents.

### Dietary Outcomes

Among the studies with a control group (*n* = 16), only nine measured dietary outcomes. Tools to measure dietary outcomes varied across studies, and not all were validated (Supplementary Information 2). In these studies, some dietary improvements were reported compared to the controls. For instance, some reported were fewer calories consumption (Alvirde-Garcia et al., 2013; Padilla‐Raygoza et al., 2013), better-eating patterns (Ponce et al., 2016; Safdie et al., 2013a, b), increase in daily dairy consumption (Radilla-Vazquez et al., 2019) or reduce carbohydrates consumption (Alvirde-Garcia et al., 2013; Levy et al., 2012a, b) at the end of the study. Vega et al. (2019) reported a significant (*p* < 0.05) increase in fruit and vegetable consumption among the intervention group. However, this result varied according to their baseline nutritional status. Bacardi-Gascon and Jiménez-Cruz (2012) reported an increased vegetable intake and decreased consumption of snacks containing fat and salt among the intervention group. Still, there was also a significant increase in sugar-sweetened beverages consumption among the intervention group compared with the control. Martinez-Andrade et al. (2014) found at three months a significant increase (*p* < 0.05) in vegetable consumption, significant reductions (*p* < 0.05) in sweet snacks and sugar added to drinks in the intervention group. However, intervention effects were attenuated at six months.

Among cohort studies (*n* = 13), only 3/13 (23%) studies measured dietary outcomes. Overall, significant improvements in dietary lifestyles were reported in these studies. Rodriguez-Ventura et al. (2018) reported that the frequency of unhealthy dietary patterns decreased, but only some (e.g., eating more fruits and vegetables and breakfast consumption) were significant (*p* < 0.05). Elizondo-Montemayor et al. (2014) and Cruz-Bello et al. (2019) reported some significant (*p* < 0.05) dietary improvements (e.g., increase in the reported consumption of fruits and vegetables and decrease in soft drinks consumption). However, these studies also reported a decrease in the consumption of milk and water.

### Physical Activity and Sedentary Behaviours Outcomes

Among the studies with a control group (*n* = 16), only 5/16 (31.2%) measured PA or sedentary outcomes. Tools to measure dietary outcomes varied across studies, and not all were reported as validated (Supplementary Information 2). Some studies reported statistically significant (*p* < 0.05) improvements compared to the controls, including a decrease in TV engagement (hr/day), sitting (hr/day), and an increased PA practice (Bacardi-Gascon & Jiménez-Cruz 2012; Gatica-Dominguez et al., 2019; Macias et al., 2014). Levy et al., (2012a, b) reported more children to be active compared to controls. However, this was not reported as statistically significant (*p* > 0.05). Safdie et al. (2013a, b) reported that PA’s increase among intervention groups (two different studies provided) vs control group was not significant. However, the step count was higher in one of the intervention groups than the other intervention and control groups.

Among cohort studies (*n* = 13), only 4/13 (30.7%) measured PA or sedentary outcomes. Rodriguez-Ventura et al. (2018) reported only a statistically significant (*p* < 0.05) decrease in the time spent watching TV. Saucedo-Molina et al. (2018) reported a statistically significant (*p* < 0.05) increase in PA in the total sample. However, such an increase was higher among boys. Elizondo-Montemayor et al. (2014) reported no significant change in self-reported PA practice. Balas-Nakash et al. (2010), which implemented two different PA routines in two separate groups, reported that one group (routine B) registered higher PA levels; however, this result was not sustained six months follow-up.

### Quality and Risk of Bias Appraisal

Only five (17.2%) of the studies had an overall strong quality, 12 (41.3%) a moderate quality, and 12 (41.3%) a poor quality (Table 2). Overall, 16 (55.2%) studies had a control group, and the study design was considered more robust than those without a control group. However, half of the studies with a control group (8/16) were randomised, and 2/8 RCTs were presented in an abstract. All except one were published in international journals or English language from the studies with the strongest quality. In most studies (79.8%), participants were considered somewhat likely to represent the target population. Some studies (44.8%) identified and controlled some analysis for relevant confounders. Because of the studies’ nature, in most of the studies blinding was not described or considered. However, two (6.8%) RCTs by Martinez-Andrade et al. (2014) and Levy et al. (2012a, b) described blinding as part of their methods. Data collection for anthropometric measurements did not raise any quality uncertainties since all the studies collected data according to international protocols. However, lifestyles outcome collection was very heterogeneous across studies. For instance, only 12 studies measured dietary lifestyles, and less than half of these were reported using validated tools (Supplementary Information 2). For studies measuring PA or sedentary lifestyles (*n* = 9), only six reported using validated tools, with only one specifically validated in Mexican children. Most studies (20; 68.9%) had follow-up rates of less than 60%. Only five studies (17.2%) reported a follow-up rate of over 80%. Very few studies (4; 13.0%) reported using intention-to-treat in the analysis of their data. For details on each publication risk of bias assessment, see Table 2.

**Table 2:** Quality assessment and risk of bias of included studies

**STUDY ID**	**SELECTION BIAS**	**STUDY DESIGN**	**CONFOUNDERS**	**BLINDING**	**DATA COLLECTION METHODS**	**WITHDRAWALS AND DROP-OUTS**	**OVERALL RATING**	**Funding**	**COI**
Alvirde-Garcia et al. (2013)	Moderate	Strong	Strong	Moderate	Strong	Weak	**MODERATE**	Funded by public national hospital and Metabolic Syndrome Institute	NR
Arroyo and Carrete (2018)	Moderate	Moderate	Weak	Moderate	Moderate	Weak	**WEAK**	No funding obtained	NR
Bacardi-Gascon and Jiménez-Cruz (2012)	Moderate	Strong	Weak	Moderate	Moderate	Strong	**MODERATE**	Funded by a public university	NR
Balas-Nakash et al. (2010)	Weak	Moderate	Strong	Moderate	Strong	Moderate	**MODERATE**	NR	Nothing to declare
Benitez-Guerrero et al. (2016)	Moderate	Strong	Weak	Moderate	Strong	Weak	**WEAK**	Funded by a public university	NR
Caballero-Garcia et al. (2017)	Moderate	Moderate	Strong	Moderate	Moderate	Weak	**MODERATE**	NR	NR
Costa-Urrutia et al. (2019)	Moderate	Moderate	Strong	Moderate	Strong	Weak	**MODERATE**	Funded by a local authority	Nothing to declare
Cruz-Bello et al. (2019)	Weak	Moderate	Weak	Moderate	Moderate	Weak	**WEAK**	NR	NR
Elizondo-Montemayor et al. (2014)	Weak	Moderate	Weak	Moderate	Moderate	Weak	**WEAK**	Funded by a private university	NR
Gatica-Dominguez et al. (2019)	Moderate	Strong	Weak	Moderate	Strong	Weak	**WEAK**	Funded by food industry (*Tresmontes lucchetti Mexico*)	NR
Macias et al. (2014) (Abstract)	Weak	Strong	Weak	Moderate	Moderate	Weak	**WEAK**	NR	NR
Martinez-Andrade et al. (2014)	Strong	Strong	Strong	Strong	Moderate	Moderate	**STRONG**	Funded by a public hospital	Nothing to declare
Mejia et al. (2016) (Abstract)	Weak	Strong	Weak	Moderate	Moderate	Weak	**WEAK**	Local health authorities	NR
Padilla-Raygoza et al. (2013)	Moderate	Strong	Moderate	Moderate	Moderate	Weak	**MODERATE**	National Ministry of Education	Nothing to declare
Perichart-Perera et al. (2008)	Moderate	Moderate	Strong	Moderate	Strong	Weak	**MODERATE**	Funded by food industry (*PepsiCo*)	NR
Polo-Oteyza et al. (2017)	Moderate	Moderate	Strong	Moderate	Strong	Weak	**MODERATE**	Funded by food industry (*Nestle*) and National public University	NR
Ponce et al. (2016)	Moderate	Strong	Weak	Moderate	Strong	Weak	**WEAK**	NR	NR
Radilla-Vazquez et al. (2019)	Moderate	Strong	Strong	Moderate	Strong	Moderate	**STRONG**	NR	NR
Ramirez-Lopez et al. (2005)	Moderate	Strong	Weak	Moderate	Strong	Weak	**WEAK**	Local authorities funding	NR
Rios-Cortazar et al. (2013)	Weak	Moderate	Weak	Moderate	Strong	Weak	**WEAK**	NR	Nothing to declare
Vazquez et al. (2017)	Moderate	Moderate	Weak	Moderate	Moderate	Weak	**WEAK**	NR	NR
Rodriguez-Ventura et al. (2018)	Moderate	Moderate	Weak	Moderate	Moderate	Moderate	**MODERATE**	Science Mexican Council	Nothing to declare
Safdie et al. (2013a, b)	Strong	Strong	Moderate	Moderate	Strong	Strong	**STRONG**	Supported by the Pan American Health Organization, Program of The International Life Science Institute, Science Mexican Council, Health Ministry, and global health Research Initiative	Nothing to declare
Salazar-Vazquez et al. (2016)	Moderate	Strong	Strong	Moderate	Strong	Weak	**MODERATE**	National Funds	Nothing to declare
Saucedo-Molina et al. (2018)	Moderate	Moderate	Weak	Moderate	Strong	Weak	**WEAK**	Funded by two private foundations	NR
Levy et al. (2012a, b)	Strong	Strong	Strong	Strong	Strong	Strong	**STRONG**	Local authorities	Nothing to declare
Vega et al. (2019)	Moderate	Strong	Strong	Moderate	Strong	Weak	**MODERATE**	NR	NR
Vilchis-Gil et al. (2016)	Moderate	Strong	Strong	Moderate	Strong	Strong	**STRONG**	Public Paediatric Hospital	Nothing to declare
Zacarias et al. (2019)	Moderate	Moderate	Weak	Moderate	Strong	Strong	**MODERATE**	Science Mexican Council and public university	Nothing to declare

^*NR*^ ^not reported, *COI* conflict of interest^

Concerning the studies’ funding, nine (31.0%) did not report any funding, and only one (Arroyo et al., 2018) reported not receiving any funding for the study. Three studies (10.3%, Gatica-Dominguez et al., 2019; Perichart-Perera et al., 2008; Polo-Oteyza et al., 2017) reported receiving funding from the food industry, three more (10.3%; Costa-Urrutia et al., 2019; Ramirez-Lopez et al., 2005; Levy et al., 2012a, b) reported using funds from local authorities. Five studies (17.2%; Mejia et al., 2016; Padilla‐Raygoza et al., 2013; Rodriguez-Ventura et al., 2018; Salazar-Vazquez et al., 2016; Zacarias et al., 2019) reported using public national funding, and five more (Alvirde-Garcia et al., 2013; Bacardi-Gascon & Jiménez-Cruz 2012; Benitez-Guerrero et al.,^ 2016^; Martinez-Andrade et al., 2014; Vilchis-Gil et al., 2016) received funding from public institutions (e.g., public hospitals or public universities). One study (Safdie et al., 2013a, b) reported being supported by local, national, and international organisations. Two (6.8%; Elizondo-Montemayor et al., 2014; Saucedo-Molina et al., 2018) reported receiving funding from private institutions (e.g. private universities, insurance companies’ funds). The authors’ conflict of interest was not reported in 18 studies (62.0%) (Table 2).

## Discussion

This work systematically reviewed interventions to prevent obesity among children and adolescents in Mexico. Twenty-nine studies (presented in 43 publications) with various experimental designs, characteristics, duration, and intensities were identified after conducting a deep search across 13 databases and one search engine. Most studies (26; 89.6%) included a nutritional component, 19 (65.5%) a PA component, 12 (41.0%) a behavioural or psychological component, and only six (20.7%) included environmental changes to support obesity prevention and lifestyles improvement. Only three studies (10.3%; Costa-Urrutia et al., 2019; Safdie et al., 2013a, b; Levy et al., 2012a, b) included several components. However, only one (Safdie et al., 2013a, 2013b) was implemented for over 12 months. Overall, very few studies (17.2%) were considered to have a strong quality, and weight-related outcomes are heterogeneous across studies with or without a control group or age group. Some (12/29) measured dietary behaviours, with most showing dietary improvements. Fewer (9/29) measured and showed PA or sedentary lifestyle improvements.

The results shown in this review are like those presented in a recent systematic review of school-based obesity prevention interventions in Latin America (Chavez & Nam, 2020). Some characteristics of the studies reported as effective align with previous children and adolescent's obesity prevention evidence. Long term and sustained (≥ 1-school year) studies with multi-component studies seem to be more effective since single-component or short-term interventions have weaker evidence on obesity prevention (Summerbell et al., 2005). All the identified studies were delivered in a single set, with most (26; 89.6%) delivered in schools. School-based studies have been reported as effective in preventing obesity and improving lifestyles (Aceves-Martins et al., 2016b; Wang et al., 2015) However, the importance of multi-setting interventions for childhood obesity prevention and treatment has also been acknowledged (Wang et al., 2015). Only six of the included studies in this review (20.7%) reported changing the environment to reinforce healthy lifestyles and prevent obesity among participants, which has been recognised as an essential factor prevent or revert childhood obesity effectively (Cauchi et al., 2016).

Children’s food choices might be influenced by observing and imitating others’ behaviour, specifically parents or siblings (Mura Paroche et al., 2017). Some of the included studies in this review (12; 41.3%) reported involving other family members in the intervention activities. Few studies (9; 31.0%) were designed in the light of a model or behavioural theory. Behavioural change may happen because of alterations in variables that mediate risk factors. These mediating variables are typically considered in theories or models used to understand behaviour (Baranowski et al., 2003). Using theories or models in the design of childhood obesity prevention studies could be helpful. Understanding the environment, triggers of risk behaviours, and including close relatives in the activities might determine the effectiveness of childhood obesity prevention (Aceves-Martins et al., 2019; Loveman et al., 2015; Mura Paroche et al., 2017). The interventions' design and method might also be critical in behavioural change and health outcomes improvement. Most of the identified evidence refers to educational interventions (e.g., teaching children benefits healthy lifestyles). Knowledge at some level is a prerequisite to the intentional performance of health-related behaviours (Baranowski et al., 2003). However, knowledge might not be enough to produce a behavioural change that prevents weight gain in the long term. For this reason, studies aiming a weight gain prevention should not be limited to educational activities.

Developing strategies to tackle childhood obesity is a complex task for different reasons: it involves several stakeholders, multiple environments need to be considered, different health risk behaviours need to be shaped, health inequalities need to be addressed, there is still an open scientific debate on the best way to address childhood obesity, and the socio-political, cultural or economic context is a critical factor that might influence the effective management of obesity (Gortmaker et al., 2015; Knai & Mckee, 2010). Nevertheless, it is noteworthy that Mexico had led to implementing different nationwide strategies to tackle obesity among the general population. For instance, a couple of years ago, Mexico introduced a 1 peso per liter tax on sugar-sweetened beverages (Colchero et al., 2017; Mostert, 2017). More recently, a front-of-pack labelling law has also been introduced (Kaufer-Horwitz et al., 2018). Additionally, in 2017, the Caribbean Public Health Agency, the Pan American Health Organisation and the Caribbean Community brought together crucial stakeholders from Mexico and Chile to develop a roadmap to prevent childhood obesity (Caballero et al., 2017). Still, effective and targeted strategies are needed urgently to avoid and revert excess weight gain among children and adolescents.

A multi-component and multidisciplinary intervention that includes dietary modifications, physical activity practice, behavioural strategies, and active youth and parental involvement might help treat childhood obesity in Mexico (Aceves-Martins, 2021a, c, 2019). Indeed, these characteristics would also benefit prevention efforts. However, prevention would usually involve complex and multifaceted health promotion efforts at a community level, which cannot be expected to produce changes in outcomes within a short time frame, especially weight outcomes. Instead, a range of effects, including attitudes and health behaviours, can be achieved (Rosen et al., 2006), affecting weight outcomes in the long term. For this reason, longer-term measurements and follow-up of participants is needed.

Retrieved evidence arises from 13 (out of 32) states. As shown in Fig. 2, most evidence comes from Mexico’s south-central area. Caballero-Garcia et al. (2017) was the only multi-site study (including children from four different states of Mexico) and reported variability of the results depending on the site. For instance, BMI reduction was different across sites. The results of such a study are discussed, considering various difficulties of running a multi-state intervention in Mexico. It is unclear the reason for such disparity of interventions identified across the different country regions. However, a considerable amount of evidence (13/29) was identified in Mexico City (the capital) and the State of Mexico, two of the most densely populated areas in the country and several universities, speciality clinical centres, and research centres can be found. Efforts to identify vulnerable populations nationwide and implementation barriers among different populations are needed as a national strategy. Also, implementing long term nationwide studies that consider and include vulnerable children or adolescents from all the regions of Mexico is essential to change the upward trends in obesity prevalence.

We found some limitations and challenges while conducting this systematic review. One of the major problems faced was the inadequate and insufficient description of methods across the included studies. Also, the lack of high-quality RCTs is noticeable. Only 7/29 (24.1%) studies were RCT and only three with strong quality. There is still a debate on the best ways to evaluate and assess the effectiveness of health promotion efforts (Rosen et al., 2006). No single method can be used to answer all relevant questions on health promotion or public health challenges (Armstrong et al., 2011). However, to evaluate the effectiveness of interventions, strong quantitative methods approaches are needed. Some suggest that randomised designs are appropriate for community-based health promotion research within the obesity prevention context (Rosen et al., 2006). Overall, the heterogeneity of the included studies, in terms of study design, sample size and characteristics, approach, follow-up length, analytical approach and overall quality of evidence, was also a limitation. The variability of these factors made not possible the effectiveness of cross-study comparison (i.e., meta-analysis), which is crucial for developing, evaluating, and improving studies, policies, practice, and research (Boaz & Davies, 2019). Also, we limited this review to those studies considering weight outcomes. By using this criterion, we might have foreseen relevant efforts to change other lifestyles that might affect long term obesity prevention.

This work’s strengths include being the first systematic review of intervention to prevent obesity in Mexican children and adolescents. An exhaustive search for evidence was done across 13 various databases and one search engine, performed in two languages, which helped us capture relevant publications. In addition, an extensive search for grey literature was conducted as part of the COMO project, but no relevant studies or interventions were identified. The characteristics of the included studies and quality appraisal were considered in the synthesis.

This review is part of a broader project aiming to synthesise and use data to comprehend the extent, nature, effects and costs of childhood or adolescent obesity in Mexico (Aceves-Martins, 2021a, b). Because of the rising levels of childhood and adolescent obesity, every effort should be considered an experiment. Effects must be documented and evaluated to benefit every other initiative or strategy. Such efforts need to enhance their methodological quality, include various settings, stakeholders, and target different health risk behaviours.

## Conclusion

Current evidence is heterogeneous and inconclusive about the efficacy of interventions to prevent obesity in Mexican children and adolescents. Overall, health promotion and prevention efforts need methodological improvement and should consider previous experiences to build evidence-based interventions. Such interventions should not be limited to educational activities and should include different components, such as multi-settings delivery, family inclusion, and longer-term implementations. Mixed-method evaluations (including strong quantitative and qualitative approaches) and follow up of participants after the intervention could provide a deeper understanding of the effectiveness and best practices.

## Supplementary Information

Below is the link to the electronic supplementary material.

Supplementary file1 (DOCX 28 KB)

Supplementary file2 (DOCX 22 KB)
