# Supplementary material for: Interventions to Prevent Obesity in Mexican Children and Adolescents: Systematic Review
Source: Prev Sci. 2021 Nov 2;23(4):563–86. doi: 10.1007/s11121-021-01316-6 (PMC9072495; doi:10.1007/s11121-021-01316-6)
Supplement: Supplementary file 1 — Supplementary file1 (DOCX 28 KB) [file 11121_2021_1316_MOESM1_ESM.docx]

**Appendix 1. Details on the interventions included in the Systematic Review.**

| **STUDY ID** | **Nutritional component** | **PA component** | **Behavioural or psychological component** | **Environmental changes** | **Other relevant components** | **Material Provided** | **Control group activities** |
| --- | --- | --- | --- | --- | --- | --- | --- |
| Alvirde-Garcia 2013 | Changes in school curricula modification | Organised activities during recess + reinforcement of PE classes + Extracurricular actions (i.e., exercise with the family). | NR | Changes in the school canteen to change food availability and quality | Family activities included + Teachers also received a lifestyle intervention for themselves | Five adapted textbooks (one per academic year) and three working books | Received a talk on childhood obesity, but school curriculum or activities were not changed. |
| Arroyo 2018 | Education on healthy eating, nutrition, and its relation to comorbidities. Discussions on risk cases with a nutritionist or teacher. | NR | Auto efficacy techniques. | NR | Parents were optionally involved. | NR | NA |
| Bacardi-Gascon 2012 | Sessions encouraging healthy eating in children, children's dietary intake were assessed, and the child's nutritional report was sent to parents at baseline and end of the intervention. | Sessions to encourage children to enrol in sports and play activities and lower the number of hrs of TV viewing. | NR | Meetings with teachers and parents improved meals and snacks offered in the school and improved PA installations and activities during and after school hrs. | NR | NR | Unclear. However, after six months, the control group started receiving the intervention. |
| Balas-Nakash 2010 | NR | Children in routine A received 20 mins of fewer intensity activities, and routine B received 40 mins of aerobic exercises. Both routines included an initial phase with warm-up exercises, a middle-phase with aerobic exercises and an ultimate phase for relaxation, following national guidelines including (30 min). | NR | NR | NR | NR | NA |
| Benitez-Guerrero 2016 | Education sessions were delivered, including topics on nutrition. | Education sessions were delivered, including topics on PA. | NR | NR | NR | NR | There was a difference between the programmes provided for the intervention and the control group. However, the difference is unclear from the reported data. |
| Caballero-Garcia 2017 | Ten educational sessions were delivered on healthy eating. | NR | NR | NR | Ten educational sessions were delivered on dental health and hygiene. | NR | NA |
| Costa-Urrutia 2019 | Workshops on healthy eating. Three meals were provided: breakfast, a snack for mid-morning playtime, and lunch at school. Diet recommendation (fat intake of 25–35%, a carbohydrate intake of 45–65% and a protein intake of 10–30%), while total calories were adjusted according to age. Beverages comprised milk in the morning and water in the rest of the servings. | PA classes comprised moderate-vigorous activity, five days/wk. PA was divided into two types: (a) in the school backyard for 60 mins, three times/wk, and (b) in the classroom for 45 min (15 min three times/day), two days/wk. Strength, resistance, velocity, coordination, stability, and cardiovascular activity were included. Also, pre-sport games were included. The complexity of circuits was adjusted (according to the school year) | Workshops on self-monitoring. | Lunch provision in school and changes in PA environment. | Parents were involved in the intervention and were offered workshops. | Book for children with the four key topics addressed in this program. In addition, parents received a report of the program and a guideline book linked to that given to children. | NA |
| Cruz-Bello 2018 | The themes of the sessions included healthy eating, concepts and differences between nutrition and food (7 sessions); feeding habits and schedules (4 sessions); Good Eating Dish, quantity, and quality (7 sessions); healthy snacks, theory, and practice (5 sessions); calorie level and quantity (5 sessions). | The session included the importance of PA (one session) and PA (3 open classes). | Eating disorders, concepts, and risk factors sessions were delivered (4 sessions); types of eating disorders, complications, and prevention (4 sessions). | NR | NR | NR | NA |
| Elizondo-Montemayor 2014 | Information shared through social media, such as healthy diet information. Also, some conferences were provided with healthy lifestyles information. | Information shared through social media, such as PA tips. Some conferences were also provided with healthy lifestyles information, and an open class of Zumba and cardio dance were offered (but not mandatory). | NR | NR | Social media was used to promote healthy lifestyles. | NR | NA |
| Gatica-Dominguez 2019 | Diet components included. However, there are not described in the paper. | Activities targeted to increase awareness among PA teachers of the quality of classes in terms of time spent on moderate or vigorous activity. Active workshops were delivered for children on the benefits of practising PA playfully. Some workshops included parents. Also, this intervention established collaborative links between local authorities and PA teachers for community sports events. There was a summer course to promote the practice of PA during the school holidays. | NR | NR | Workshops with parents (parents performed exercises and recreational PA) + Trained personnel accompanied the school community daily to stimulate children, parents, and teachers to supervise the correct implementation + holiday and extracurricular activities. | Provision of recreational materials to practice PA (Unclear from the report what type of material). | No intervention and monitoring of significant changes in the control community were delivered. Likewise, while monitoring was carried out in the schools without intervention, monitor the general conditions and record any critical changes that could influence the children's PA in the control community. |
| Macias 2014 (abstract) | Unclear | Unclear | NR | NR | NR | NR | Traditional education group received one session/wk. |
| Martinez-Andrade 2014 | Educational sessions for parents and children on healthy dietary behaviours were delivered. In addition, socialising and building group rapport through activities like preparation and consumption of healthy foods on-site were offered. | Educational sessions for parents and children on PA were delivered. The nurse provided childcare and developed relevant games and activities for children while parents attended the workshops. | Educational sessions employed motivational interviewing and reflexive listening techniques to enhance self-efficacy. | NR | Parents and siblings were included in the intervention. | Illustrated manual and recipe book for parents, and a children's card game "memory" with pictures of foods were provided. In addition, transportation costs were reimbursed to participants. | The usual standard of care clinical practice guide within clinic guidelines, children, may be referred to a nutritionist if the physician considers it appropriate (but is not mandatory). |
| Mejia 2017 (Abstract) | Nutrition education was delivered (no further detail was provided). | Daily PA practice was delivered (no further detail was provided). | NR | Lunch provision in school. | Intervention targeting teachers and parents. | NR | Children in the control group had no intervention. |
| Padilla-Raygoza 2013 | Teaching selection of healthy food and preparation of meals to the mother of the children was delivered. | 30 mins of walk in the school from Monday to Friday was performed. | NR | NR | NR | NR | NR |
| Perichart-Perera 2007 | Teachers provided food orientation and PA messages to all schoolchildren during their school hrs (min 30 mins/wk) to promote a correct energy balance. | A 20-minute exercise routine was designed, following the national guidelines. The routine included a warm-up phase (3-5 mins), a flexibility, strength, and balance phase (5-10 mins) and a relaxation phase (six mins). This routine was compulsory for all schoolchildren who attended schools before the morning start + Teachers provided food orientation and PA messages to all schoolchildren during their school hrs (min 30 mins/wk) to promote a correct energy balance. | NR | NR | NR | NR | NA |
| Polo-Oteyza 2017 | No | Teachers were trained. Activities allowed free exchange between peers and teachers. Two or three days per wk, brisk walking was substituted with play and games. | NR | NR | No attempt to modify other factors, such as diet or extracurricular activities, was made. | NR | NA |
| Ponce-y-Ponce-de-Leon 2016 | Educational sessions included healthy diet sessions, healthy eating, and its relationship with a healthier life. Sessions briefly explained the topic to be discussed. With active individual and team participation, knowledge was built through various dynamics, finally getting the individual preparation of a healthy menu. | NR | NR | NR | NR | NR | NR |
| Radilla-Vazquez 2019 | Nutritional orientation was given with multidisciplinary work and training of teachers and school doctors. | Unclear | Unclear. There was a psychology practitioner involved, and the intervention was multidisciplinary. | NR | Parents and teachers (School doctors, science, and physical education teachers) were trained. | Twenty-four printed comics were as educational materials. | Digital materials were used, 24 comics were available online. Also, nutritional counselling was given without multidisciplinary work. |
| Ramirez-Lopez 2005 | Provision of breakfast from a governmental programme. | NR | NR | NR | Breakfast provision. | NR | No breakfast was provided. |
| Rios-Cortazar 2013 | Collective production of children's stories, later reflected in a monthly school newspaper, collects, and reflects the experiences related to their diet, health, and nutrition. | Collective production of children's stories, later reflected in a monthly school newspaper, collects, and reflects the experiences related to their PA. | NR | NR | NR | The newspaper was created with views of children. | NA |
| Rivera-Vazquez 2016 | The educational session, including a poster and delivery of diptychs using the active method, was delivered, allowing the understanding, debate, and conclusions healthy lifestyle. | NR | NR | NR | NR | Printed material with healthy lifestyles was provided. | NA |
| Rodriguez-Ventura 2018 | Workshops included guidance on food groups, portion sizes of each food group using replica models, healthy and unhealthy combinations of food groups, examples of healthy menus and meal plan explanations. In addition, all the participants received personalised dietary plans designed by a nutritionist. | All the patients received a pedometer and complimentary messages on healthy lifestyles during the workshops. | Strategies to improve lifestyle habits organising schedules, awareness, working as a family and empowering their individual decisions. | NR | Family active participation. | NR | NR |
| Safdie 2013 | The nutrition intervention aimed to improve the prevailing food environment by increasing the availability of healthy food (fruits, vegetables, and non-fried dishes) and beverages (particularly water) by reducing energy-dense foods and sugary drinks. | The PA intervention aimed to enhance the prevailing PA environment by increasing PA resources' availability by improving infrastructure and enhancing aesthetics. In plus schools only, specialised physical education teachers were hired to teach one additional class per wk and offer 15 to 20 mins of moderate PA four days/wk. | The social cognitive theory was used to increase student and school staff awareness of program activities and develop positive PA and healthy eating attitudes at school planned behaviour. | Only the plus programme. The plus program implemented all the components incorporated in the primary program and included additional financial investment and human resources. | NR | NR | NR |
| Salazar-Vazquez 2016 | Intervention not targeted to change dietary patterns. However, participants and parents were shown the weight by age, also promoted home-cooked meals. Recommendations such as: possibly avoiding sugary drinks; do not talk and eat at the same time; no repeated portions; no overfilling; no eating or snacking between meals; and no eating of the table was given. | NR | Behavioural counselling (specific to the eating process) Additional recommendations are given eat slowly, using the hourglass as a guideline. | NR | Participants were also instructed on the use of the 30-s hourglass to pace bites during meals. They received a guide with recommendations on how to follow the programme. | 30-s hourglass. | The control group did not receive any instructions. The control group and their parents were fully informed of the programme on purpose and agreed to be measured according to the programme's schedule, at which time they received heath advice. |
| Saucedo-Molina 2018 | Educational sessions and activities on healthy eating were delivered. | Four sessions included enjoyable PA. This hr of PA took place inside or outside the classroom, depending on the weather. | Critique session and discuss the body ideals promoted through advertising. Students were also invited to write a letter to dissuade a friend or family member who engages in certain risky behaviours. | NR | NR | NR | NR |
| Shamah-Levy 2012 | Campaign to promote consuming one fruit and one vegetable, drinking pure water was implemented. There were nutrition workshops with recreational activities to handpick healthy foods. Parents received a recipe calendar with healthy recipes for school lunches. | A campaign to promote PA was implemented. There were PA workshops with recreational activities for children to gain knowledge and skills. Teaching resources were included. Parents received a recipe calendar with PA activities. There were organised activities involving motion games during the break (once per wk). Educational materials were provided for these activities, including posters with suggestions for team games and activities that involved moving during 30-mins breaks. | The workshop should reinforce and expand knowledge and foster self-assessment. Teaching resources were included. There was a puppet theatre based on the theory of peer learning. | The gradual regulation of food offered within the school was introduced through Mexico's technical council. Activities to change the school environment include selling fruits, vegetables, and pure water in the school's store cooperative. Spots were broadcast three times per wk during the break using the school's audio systems, and water bottles were delivered to children and teachers to encourage water consumption. In addition, a banner was hung that read, "This school promotes healthy breaks." | Teachers, parents, and personnel from school stores also received a comprehensive intervention. | Student booklets and a facilitator's guide; a school guide; a calendar for parents, as well as videos (or printed handouts for schools with no DVD players) and audio spots. Recipe calendars for parents. | No intervention. |
| Vega-y-Leon 2019 | Food orientation educational sessions included topics such as healthy eating. | NR | Psychoeducational workshops were delivered (no further information). | NR | Parents and teachers were involved. | Printed educational materials were provided. | No nutritional guidance was given. |
| Vilchis-Gil 2016 | Health promotion workshops and website material on nutritional status and healthy eating were provided. | Health promotion workshops and website material on PA and sedentary lifestyles were delivered. | Health promotion workshops and website material on self-monitoring, family behaviour and integration of all the intervention messages were delivered. | NR | Parents involved + website support + text messages to parents + visit to children and parents visited the Life in Balance room of the Science Museum + after each anthropometric measurement; children were handed a letter with the results of the nutritional status and tips to maintain or improve their health. | A website with materials was available. The artworks for this project, such as images of children eating healthy foods, were created by the designers who considered the age of the children culture of Mexico City. A paediatric nutrition expert edited the topic. Information posters were placed in strategic locations and frequently changed within the intervention schools. Various materials were developed for the children to take home. | NR |
| Zacarias 2019 | Educational sessions to mothers, including healthy habits, healthy weight for children and healthy eating, were delivered. | NR | The Social Cognitive Therapy construct used in each specific session was provided. | NR | Mother included in the activities. In addition, a financial workshop for mothers was provided. | A booklet with graphical material of the sessions and recipes considering the cultural background was provided. | NR |

PA= Physical Activity, wk=week, mins= minutes, hour=hr, NA=Not Applicable, NR= Not Reported
