# Supplementary material for: Interventions to Prevent Obesity in Mexican Children and Adolescents: Systematic Review
Source: Prev Sci. 2021 Nov 2;23(4):563–86. doi: 10.1007/s11121-021-01316-6 (PMC9072495; doi:10.1007/s11121-021-01316-6)
Supplement: Supplementary file 2 — Supplementary file2 (DOCX 22 KB) [file 11121_2021_1316_MOESM2_ESM.docx]

**Appendix 2. Overview of relevant outcomes**

| **STUDY ID** | **Diet-related outcomes** | **Validation** | **PA or sedentary behaviours related outcomes** | **Validation** |
| --- | --- | --- | --- | --- |
| **Alvirde-Garcia 2013**  Randomised Control Trial | Decrease in calorie intake during school hours and reduced sugar and fat consumption in the intervention group compared with the control group. | Unclear | NR | NA |
| **Arroyo 2018**  Cohort (one group before and after) | At the end of the intervention, a more significant percentage of teenagers perceived themselves as self-efficient to choose their food than baseline measurements. | No | NR | NA |
| **Bacardi-Gascon 2012**  Randomised Control Trial | At the end of the study, there was a significant increase in vegetable intake and decreased fat and salty snacks. However, there was also a significant increase in the consumption of sugar-sweetened beverages. | Unclear | TV engagement (hr/day) and sitting (hr/day) decreased significantly. Physical Education (hr/wk) and supervised sports or dance (hr/wk) increased significantly. | Yes, survey validated in Chile (INTA PA questionnaire) |
| **Balas-Nakash 2010**  Cohort analytic (two groups before and after) | NR | NA | More children in routine B achieved the aerobic exercise threshold of 120 beats/min and worked at a higher %HRmax. However, after six months of intervention, all children reported the same sedentary activity levels, and no differences were observed between groups. | Yes, a questionnaire validated in the Mexican population |
| **Benitez-Guerrero 2016**  Controlled trial | NR | NA | NR | NA |
| **Caballero-Garcia 2017**  Cohort (one group before and after) | Although measured, no effect or changes in the dietary pattern were reported in the publication. | Unclear | NR | NA |
| **Costa-Urrutia 2019**  Cohort analytic (two groups before and after) | NR | NA | NR | NA |
| **Cruz-Bello 2018**  Cohort (one group before and after) | Increase of the number of meals, reported water consumption, decreased the consumption of soda or flavoured water, reported fruit and vegetable consumption, cereals, and tubers and, to a lesser extent, consumption of food of animal origin. | Unclear | NR | NA |
| **Elizondo-Montemayor 2014**  Cohort (one group before and after) | There was a significant increase in the reported consumption of fruits and vegetables. Also, there was a decrease in the reported consumption of soft drinks, chips, milk, and water. | No | No change in self-reported PA practice was stated. | No |
| **Gatica-Dominguez 2019**  Controlled trial | NR | NA | An important limitation of this study is not having the baseline measurement of physical condition before the intervention. However, most PA measures (e.g., 6 mins walking test or 50 metres speed test) are reported to be better for the intervention group than the control group at the end of the intervention. | Yes, walk test in six mins & 50-meter flat test & pedometer adapted for children. |
| **Macias 2014 (abstract)**  Randomised Control Trial | NR | NA | Mins/wk of PA practice were significantly increased in the intervention group compared to the control. | Unclear |
| **Martinez-Andrade 2014**  Pilot - Randomised Control Trial | At three months, the intervention group significantly increases vegetable consumption and reductions in sweet snacks and sugar added to drinks. However, at six months, these potential intervention effects were attenuated. | Yes, the Food Frequency Questionnaire (FFQ) adapted from the FFQ that assess dietary intake among 1-4-year-olds in the 2006 Mexican National Nutrition Survey | NR | No |
| **Mejia 2017 (Abstract)**  Randomised Control Trial | NR | NA | NR | NA |
| **Padilla-Raygoza 2013**  Randomised Control Trial | After four months, the intervention group reported consuming fewer calories than the control group. | NA | NR | NA |
| **Perichart-Perera 2007**  Cohort (one group before and after) | NR | NA | NR | NA |
| **Polo-Oteyza 2017**  Cohort (one group before and after) | NR | NA | NR | NA |
| **Ponce-y-Ponce-de-Leon 2016**  Controlled trial | After the intervention, the intervention group reported better and healthier eating patterns than the control group. | Yes, Krece Plus nutrition test for the population aged 4 to 14 years to identify eating habits. | NR | NA |
| **Radilla-Vazquez 2019**  Controlled trial | There was an increase in the daily consumption of dairy in adolescents in the intervention group. In the intervention group, it was found that adolescents who never consume whole milk, fresh cow cheese and plain yoghurt have a higher prevalence of obesity than adolescents who consume them daily. In addition, a positive association was observed in the consumption of skim milk with nutritional status. However, this is because, at the end of the intervention, adolescents with overweight or obesity increased their consumption of this type of dairy more, with a highly significant difference. In the control group, it was also observed that the higher the consumption of dairy products, the lower the percentage of obesity. | Yes, 24-hour food recall and a frequency of food consumption questionnaire. | NR | NA |
| **Ramirez-Lopez 2005**  Controlled trial | NR | NA | NR | NA |
| **Rios-Cortazar 2013**  Cohort (one group before and after) | NR | NA | NR | NA |
| **Rivera-Vazquez 2016**  Cohort (one group before and after) | NR | NA | NR | NA |
| **Rodriguez-Ventura 2018**  Pilot - Cohort (one group before and after) | The frequency of unhealthy dietary habits decreased, but only some (e.g., eating more fruits and vegetables and breakfast consumption) were significant. Calorie’s consumption was reported to be significantly lower at the end of the intervention. | Yes, 24-hour food recall and a frequency of food consumption questionnaire. | Reported time on watching TV was the only lifestyle that significantly was reduced. | Unclear |
| **Safdie 2013**  Randomised Control Trial | There were significant changes in the distribution of food available among the three categories of food over the two years of intervention characterised by an increase in the percentage of the highly recommended food and reduced the percentage of non-recommended food items in both interventions to control schools. In addition, potable drinking water was available to some of the intervention's schools by the end of the intervention. Overall, the children's food intake changes were notable across most of the assessments, but not all were significant. | Yes, food inventories. | Changes in moderate to vigorous PA in children during physical education classes and recess were not significant. Steps taken increased significantly only in the primary intervention group; however, the plus group showed an increasing tendency but was not significant, and the control group decreased the steps count. | Yes, SOFIT (System for Observing Fitness Instruction Time) + pedometer. |
| **Salazar-Vazquez 2016**  Controlled trial | NR | NA | NR |  |
| **Saucedo-Molina 2018**  Pilot - Cohort (one group before and after) | NR | NA | There was a significant increase in PA in the total sample, which was higher in boys. | Yes, the International PA Questionnaire (IPAQ) using the version validated in Mexico. |
| **Shamah-Levy 2012**  Randomised Control Trial | The intervention group showed a lower risk of overweight associated with the combined interaction effect of the intervention and carbohydrates' consumption. | Yes, Food Frequency Questionnaire. | More children were active in the intervention group than in the control group; however, this was not significant. | Yes, the Youth activity questionnaire. |
| **Vega-y-Leon 201**  Controlled trial | After the intervention, there was a significant increase in fruit and vegetable consumption. However, this result varied on nutritional status. | Yes, the Food Frequency Questionnaire | NR | NA |
| **Vilchis-Gil 2016**  Controlled trial | NR | NA | NR | NA |
| **Zacarias 2019**  Cohort (one group before and after) | NR | Yes, Food Frequency Questionnaire. | NR | Yes, PA frequency. |

NR=Not Reported, NA= Not applicable, min=minutes, hr=hours, PA=physical activity, BMI= Body Mass Index
